# Microglia and Astrocytes Dysfunction and Key Neuroinflammation-Based Biomarkers in Parkinson’s Disease

**DOI:** 10.3390/brainsci13040634

**Published:** 2023-04-07

**Authors:** Kun Chen, Haoyang Wang, Iqra Ilyas, Arif Mahmood, Lijun Hou

**Affiliations:** 1 Department of Neurosurgery, Changzheng Hospital, Naval Medical University, Shanghai 200003, China; 2 National Centre of Excellence in Molecular Biology (CEMB), University of The Punjab, Lahore 53700, Pakistan; 3 Center for Medical Genetics and Hunan Key Laboratory of Medical Genetics, School of Life Sciences, Central South University, Changsha 410078, China

**Keywords:** Parkinson’s disease, astrocytes, microglia, biomarkers, glial cells, drug development

## Abstract

Parkinson’s disease (PD) is the second most common neurodegenerative disease, with symptoms such as tremor, bradykinesia with rigidity, and depression appearing in the late stage of life. The key hallmark of PD is the loss or death of dopaminergic neurons in the region substantia nigra pars compacta. Neuroinflammation plays a key role in the etiology of PD, and the contribution of immunity-related events spurred the researchers to identify anti-inflammatory agents for the treatment of PD. Neuroinflammation-based biomarkers have been identified for diagnosing PD, and many cellular and animal models have been used to explain the underlying mechanism; however, the specific cause of neuroinflammation remains uncertain, and more research is underway. So far, microglia and astrocyte dysregulation has been reported in PD. Patients with PD develop neural toxicity, inflammation, and inclusion bodies due to activated microglia and a-synuclein–induced astrocyte conversion into A1 astrocytes. Major phenotypes of PD appear in the late stage of life, so there is a need to identify key early-stage biomarkers for proper management and diagnosis. Studies are under way to identify key neuroinflammation-based biomarkers for early detection of PD. This review uses a constructive analysis approach by studying and analyzing different research studies focused on the role of neuroinflammation in PD. The review summarizes microglia, astrocyte dysfunction, neuroinflammation, and key biomarkers in PD. An approach that incorporates multiple biomarkers could provide more reliable diagnosis of PD.

## 1. Introduction

Among neurodegenerative diseases, Parkinson’s disease (PD) is the second most common after Alzheimer’s disease, affecting 2% of people worldwide. In the United States, Europe, and surrounding countries, the prevalence of PD is high, including in some Asian countries [1]. The progressive degeneration of dopaminergic neurons that occur in the substantia nigra pars compacta (SNpc) causes motor impairment in PD. The mechanism of this neurodegeneration is still largely unknown, but many genetic and environmental factors have been associated with disease occurrence and pathogenesis [2]. Genome-wide association studies (GWAS) suggest that the dysregulation of innate and adaptive immunities may be a key contributor in PD pathogenesis [3]. Several pathoetiological mechanisms contribute to neurodegeneration, including sustained inflammatory processes [4]. The progression of neurodegenerative disorders is typically associated with chronic inflammation as opposed to acute inflammation, which is usually associated with repairing the brain after exposure to a variety of environmental insults such as viral infection, traumatic injury, and toxins [5]. The association of these inflammatory processes with PD are not well defined; however, polymorphism and genetic variants of genes related to immunity have been reported [6,7]. Additionally, animal models, neuroimaging, and postmortem pathology have provided detailed insight into the role of inflammation in PD pathogenesis and suggest that cytokine-induced inflammation may plays a crucial and vital role [8].

Several factors including hypoxia/ischemia, peripheral nerve injury, trauma (physical and psychological), toxins, and infection activate glial cells in the central nervous system (CNS) [9,10,11]. Physical and chemical support to neurons is provided by glial cells, which also maintain their environment. Neurons communicate with different glial cell in order to properly perform functions [12]. Among many glial cells, mostly microglia and astrocytes play a phagocytotic role by engulfing synapses and apoptotic cells and releasing toxins. Microglia are the resident macrophages of the brain that represent 5–10% of the whole CNS and are necessary for CNS homeostasis [13]. In the brain, glia cells constitute over 50% of all the cells and are divided into several types, including astrocytes. Over 100 years have passed since the discovery of astrocytes, but not much information is available about their functions in neurological diseases. Astrocytes have key functions in the brain, including an active role in circuit building, synaptic turnover, and ion homeostasis. “Astrocyte reactivity”, “astrocyte activation”, “astrogliosis”, “astrocytosis”, and “reactive gliosis” are terms that describe different morphological, molecular, and functional changes in astrocytes [14]. Recent studies suggest that mitochondria plays an important role in the regulation of astrocyte functions such as transmitophagy, calcium signaling, antioxidant production, glutamate regulation, fatty acid metabolism, and neuro-inflammatory activation, which indicates that mitochondrial dysfunction in astrocytes may affect DA neuron health, consequently leading to the death of these neurons [15,16]. Furthermore, rapamycin (modulator of autophagy and inducer of mitophagy) and bafilomycin (mitophagy inhibitor) induce changes in several glial targets that regulate development, differentiation, and astrocyte survival [17,18]. Such molecular targets include those proteins that regulate the activation of astrocytes in the course of neuroinflammation and control anti-inflammatory and proinflammatory phenotypes. The evidence obtained from several studies suggested that astrocytes play a central role in PD pathophysiology.

A major pathophysiological factor in PD is neuroinflammation in the SNpc caused by astrocyte reactivity. PD etiology has also been associated with expression of several PD-related genes by neurons and glia cells, suggesting that mutated gene products within microglia and astrocytes might play a role in PD progression [19,20]. Many neurodegenerative disorders, including PD, multiple system atrophy (MSA), dementia, and progressive supranuclear palsy (PSP), share common clinical characteristics, which makes the diagnosis more challenging [21]. Understanding neuroinflammation in context with microglia and astrocytes will provide more insight into the mechanism. The identification of key neuroinflammation-based biomarkers can facilitate early detection of PD.

### Role of Inflammation in PD

In the 1980s for the first time, activated microglial infiltration in the substantia nigra of the postmortem PD brain was observed by McGeer [22]. Numerous studies have concluded that PD pathogenesis is associated with neuroinflammation caused by cytokines secreted by activated microglia, including IL-6, interleukin B (IL-1B), and tumor necrosis factor-α (TNF-a) [23,24,25]. Inflammation and immune dysfunction are closely associated in PD. Several studies conducted with peripheral blood and cerebrospinal fluid (CSF) collected from PD patients suggested that any alteration in inflammation markers and immune cells leads to initiation and exacerbation of neuroinflammation and continues the neurodegenerative process [26]. In addition, inflammation in both the central and peripheral regions of the brain are associated with PD pathogenesis. Central inflammation involves T cells, microglia, and astroglia within the PD CNS, and peripheral inflammation involves T cell signaling and activation of innate cells in the entire CNS, the gastrointestinal (GI) tract, and blood [2]. 

In addition, some studies have used microglial activation-based radiotracers to understand and record neuroinflammation events in PD [27]. PD pathogenesis is also influenced by humoral adaptive immunity. Several autoantibodies target CNS-specific proteins, including tau, neurofilament, glial fibrillar acidic protein (GFAP), S100B, and neuronal calcium channels; additionally, autoantibodies to brain protein α-synuclein have also been discovered. PD is characterized by higher levels of antimelanin antibodies in the blood. Collectively, these discoveries suggest that both adaptive and innate immunity are triggered in PD. 

A multiprotein complex called an inflammatory response complex (inflammasome) is primarily responsible for sensing internal stress signals such as environmental, metabolic, and cellular signals. AIM2, NLRP1, NLRP2, NLRP3, NLRP4, and NLRP5 are multiple inflammasomes expressed in immune, glial, and neuronal cells of the CNS. NLR-family proteins correspond to a wide range of biological stimuli, including misfolded protein aggregates, bacterial components during CNS infection, and viral DNA. Inflammasomes belonging to the NLR pyrin domain-containing 3 (NLRP3) family are the most extensively studied in PD, containing the signaling adapter ASC and the caspase-1 enzyme [28]. As misfolded alpha-synuclein accumulates in the brain, persistent NLRP3 inflammasome activation results in dopaminergic (DA) neuronal cell death through proinflammatory cytokines. Inflammasomes drive the pathology and further propagation of protein aggregates in the CNS that are activated by chronic inflammasome activation [29].

## 2. Microglia in PD

Microglia are macrophages residing in the CNS and are the primary immune cells. Microglia make up about 10% of the cellular population in a healthy and nondiseased human brain [30]. In the physiological state, microglia mediate several brain functions, primarily synaptic pruning and remodeling and bidirectional signaling, and are important for neural circuits and brain connectivity [31]. Pathological triggers initiate the migration of microglia to the area of injury, where they act as a double-edged sword to either relieve or aggravate the injury. Interestingly, activation of microglia is related to neurodegeneration, the process underlying PD and many other neurodegenerative diseases. 

Recently, microglia in PD have drawn prominent attention because of their role as regulators of immunity, primarily in initiation of neuroinflammation in response to proinflammatory molecules. A recent discovery and breakthrough suggest that both the innate immune system and microglia are important for synaptic lopping which describes their contribution in imparting changes to the neurons that surrounds them [32]. The microglial population undergoes controlled cycles of renewal, which maintains their appropriate overall density and may also modulate the relative proportions of different microglial phenotypes [33]. In this way, microglia play a crucial role in maintaining parenchymal homeostasis by being mobile, dynamic, and vigilant observers [34]. Microglia-derived inflammation plays a role in provoking astrocytes to attain neurotoxic functions or lose neurotrophic or synaptoptrophic functionality [35]. Microglia normally maintain functional nerve cells by synaptic pruning, while dysfunctional microglia during synapse phagocytosis led to synaptic dysfunction and neurodegeneration [31]. Moreover, microglia also perform an important function in the clearing of necrotic and apoptotic cells and, further, abolishing aberrant and toxic protein clumps including alpha-synuclein aggregates and β-amyloid [36,37]. Generally, microglia participate in CNS autophagy using phagocytosis and maintaining homeostasis by eliminating the effect of inflammatory response. Microglial autophagy refers to the transport of damaged toxic organelles and protein aggregates to the lysosome by a cascade of events such as autophagosome formation and protein degradation [38]. The dysregulation of autophagic flux in the autophagy pathway hence affects overall the autophagy pathway and which is the major contributor to the PD. 

**Figure 1:**
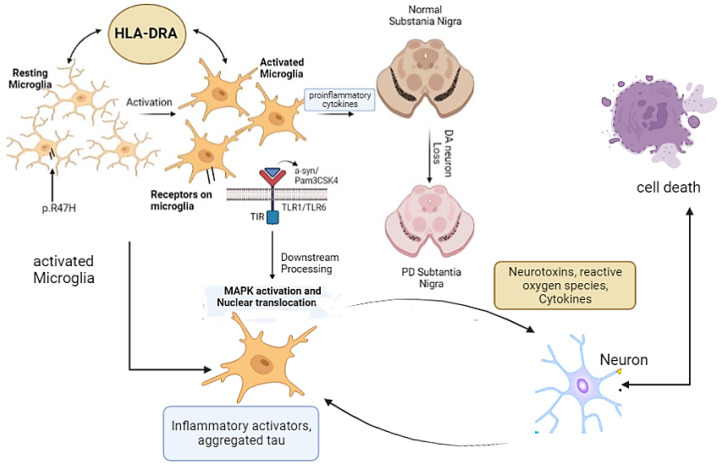
Microglia and microglial receptors in PD. Resting microglia are activated by different stimuli, which ultimately activate MAPK and cell death. Activation of microglia alternatively leads to neuroinflammation and loss of DA neurons and causes PD.

The neuroinflammation in PD is associated with microgliosis. A 2013 genome-wide association study (GWAS) first showed the involvement of the leukocyte antigen gene (HLA-DRA) in the neuroinflammation, which has specifically high expression in microglia [39]. The variant of microglia-triggering receptors (p.R47H), which is expressed on myeloid cells-2 called TREM-2, is associated with PD [10]. In the inflammatory mechanism of PD, TREM2 can affect microglial activation and autophagy through the mTOR/p38 MAPK pathway and thus affect pathological changes in PD [40], as MAPK1 pathway is highlighted in Figure 1. In addition to this, positron emission tomography (PET) studies found that reactive microglia are detected in toxin-induced and transgenic mouse models with PD [41,42]. These findings suggested that microglial activation is correlated with the PD progression and that the microgliosis induces DA neuron (midbrain dopaminergic neuron) toxicity and death.

Microglia receptors in the CNS are mostly pattern-recognition receptors (PRRs) that respond to pathogen-associated molecular patterns (PAMPs), one of the prominent types in toll-like receptors (TLRs). Studies showed that microglial receptor TLR1/6 and its downstream pathways enable neuroinflammation by microglia [43,44]. Ligands such as α-Syn and Pam3CSK4 activate TLR2-mediated downstream signaling via the adaptor coreceptor, either TLR1 or TLR6 [35]. Upon α-Syn activation, interaction between the TIR domain activates the kinase activity of the interleukin-1 receptor-associated kinase (IRAK) complex, which then interacts with and activates the TNF receptor-associated factor 6 (TRAF6) via its K63-linked autoubiquitination [33]. This process leads to activation of the transforming growth factor β-activated kinase-1 (TAK1) complex, which then mediates the IκBα degradation. These sequential events lead to the production of proinflammatory cytokines through MAPK activation and the nuclear translocation of NF-κB and p3 [43,45].

The results of the primary microglial culture from the mice treated with the human alpha-synuclein aggregate revealed that alpha-synuclein can directly interact with microglia and can be internalized and transported to autophagosomes. This interaction is mediated by FcγRs, and when the gamma chain is missing, NF-B signaling, and intracellular trafficking are altered. In other studies, using rate primary cultured microglia, the authors found that alpha-synuclein can induce the expression of matrix metalloproteinases (MMPs) (MMP-1, -3, -8, and -9) [46,47]. PD risk factors are also associated with microglial activation and its progression. The activation of microglia by inducing the expression of matrix metalloproteinases and the subsequent activation of protease-activated receptor-1 are one among the main contributors in PD. Clinical and animal experimental models have suggested a notable higher level of MG during the progression of PD. There was evidence of morphological changes including enlarged and functionally stronger MG than other phagocytoses during proinflammatory response [48]. 

## 3. Astrocytes in PD

**Figure 2:**
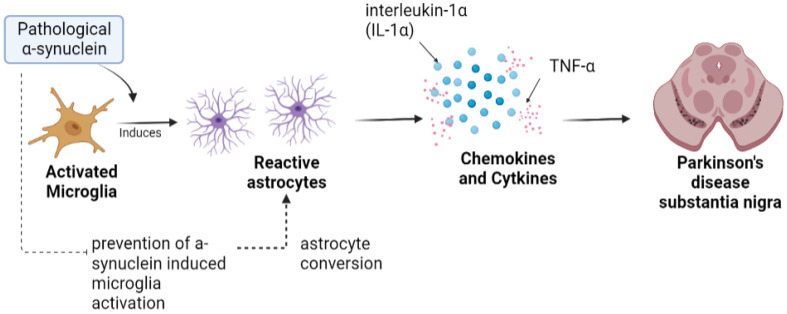
Astrocytes in Parkinson’s disease. Toxic alpha-synuclein convert resting astrocytes into reactive astrocyte, which further disrupts balance of cytokines that can possibly result in loss of DA neurons.

Astrocytes are specialized glial cells that are present in enormous numbers in the CNS, playing various physiological roles such as synaptic transmission regulation, secretion of neurotrophic molecules, and control of the permeability of the blood–brain barrier (BBB) [49]. It has been observed in PD that there is a disruption of the BBB with differentiation of dopaminergic neurons, which suggests that the normal function of astrocytes is lost with the subsequent progression of PD [50]. Reactive astrocytes are formed in response to injuries in the CNS by releasing a variety of chemokines and cytokines that include tumor necrosis factor alpha (TNF-α) and interleukin-1 beta (IL1β), the release of which aids in PD pathogenesis by toxic gain of function [51]. Another study showed that activation by microglia is induced by pathological α-synuclein, followed by induction of neurotoxic reactive astrocyte by secreting interleukin-1α (IL-1α), TNF-α. Moreover, it was observed in a mouse model of sporadic PD that pathological α-synuclein prevents α-synuclein-induced microglial activation and protects the astrocyte against dopaminergic degeneration [52], as illustrated in Figure 2.

The identification of PD progression in pathological contexts leads to the accumulation of a-synuclein-positive cytoplasmic inclusions in neurons. Alpha-synuclein is encoded by the SNCA gene, and mutation or disruption in this gene leads to PD. It has been observed that there is low expression of SNCA in astrocytes compared to its expression in neurons [53]. The protein α-synuclein plays a physiological role such as mediation in astrocytes. Its deficiency disrupts astrocyte fatty acid uptake and trafficking. Pathologic α-synuclein accumulated is evident in the postmortem brains of PD patients [54]. It has been also found that cell-to-cell transmission of α-synuclein contributes to the predominant expression of alpha-synuclein in neurons, which leads to formation of inclusion bodies in astrocytes [55,56]. The astrocytes that are accumulated with α-synuclein have shown proinflammatory cytokines such as IL-1 and IL-6 as well as some chemokines [56]. These chemokines contribute to the dysfunction of astrocytes, leading to PD progression and pathogenesis. The TLR-4 independent endocytosis pathway showed that, astrocytes can take up alpha-synuclein [57,58]. The endocytosed alpha-synuclein is localized to the lysosome, suggesting astrocyte’s role in alpha-synuclein’s degradation and removal. A high concentration of alpha-synuclein extracellularly has been shown to induce TLR4-dependent inflammatory response in primary astrocyte cultures [51]. In other words, this response is concentration-dependent; when the concentration of alpha-synuclein exceeds the threshold, then an inflammatory response is induced, and PD begins to develop, whereas in other cases when alpha-synuclein secretes from the neuron, astrocytes endocytose and degrade them [59]. 

Astrocyte dysfunction is possible due to five main mechanisms [60]: first, through the aquaporin-4 (AQP4) water channels, which are mislocalized and distant from the astrocytes, and second, through reduction in the neuroprotective capacity of astrocytes. The aggregation of alpha-synuclein leads to a decrease in the neurotrophic factor release. This damages the astrocyte function and results in decreased neuroprotection of astrocytes. In the third mechanism, astrocyte contributes to neural toxicity through inflammatory signaling via an increase in inflammasome pathways of TLR4, IFN-g, and NLPR3. In the fourth mechanism, impaired astrocyte proliferation occurs, which reduces the capacity of cells to respond. In the fifth mechanism, the reduction in glutamate uptake leads to an increase in extracellular glutamate, thus contributing neuronal excitotoxicity [54,61,62]. 

## 4. Neuroinflammation-Based Biomarkers in PD

Inflammation plays a vital role in PD pathogenesis and its symptoms can also play a critical role in diagnosis of PD; however, there are limited and irregular studies on the role of inflammation in PD. Interestingly, symptoms of PD and atypical parkinsonism (APD) overlap especially in the early disease process [63]. Patients with PD show faster disease progression and also show inefficient response to present PD treatments. Neutrophil-to-lymphocyte ratio (NLR) is also interpreted as a possible biomarker of neuroinflammation in the peripheral region, which is also useful in distinguishing between PD and supranuclear palsy [64]. Platelet-to-lymphocyte ratio (PLR) is another parameter that reflects the level of inflammation, because the platelets also play a role in peripheral inflammation [65]. Several proinflammatory cytokines, such as interleukin (IL)-1β, IL-6, and tumor necrosis factor-α (TNF-α), are secreted by activated microglia that are associated with neuronal damage, with subsequent release of the major histocompatibility complex (MHC) II [66]. Biomarkers can provide early insight into the progression of PD and treatments responses. Candidates among neuroinflammation biomarkers include α-synuclein, tau, and Aβ42 in CSF, blood, and other body fluids, which are potential sources of research interest [67,68]. As compared to the extraction of live human neurons from PD patients, cerebrospinal fluid (CSF) is a more acceptable source for measuring the molecular changes underlying neurodegenerative pathogenesis. Inflammatory molecules and peripheral blood are other sources of potential biomarkers [69]. Leakage of inflammatory biomarkers in the blood of PD patients indicates the involvement of peripheral regions such as gut–brain axis in PD pathogenesis. Potential peripheral biomarkers include IL-1β, IL-2, IL-6, IL-10, high-sensitivity C-reactive protein (hsCRP), and TNF-α/soluble TNF-receptors (sTNFRs), which are regulated upon activation [70].

Some genetic mutations are also involved in the neuroinflammation of PD. These includes Leucine-Rich Repeat Kinase 2 (LRRK2), which is a monogenic genetic cause of PD [71]; PTEN-Induced Putative Kinase 1 (PINK1), linked to familial PD with autosomal recessive inheritance [72]; PARKIN (PRKN), common in patients with early-onset PD [73]; and DJ-1, which is found in the familial recessive form of PD [74]. Some radiotracers that target the inflammatory cells via microglia activation can help in observing and monitoring the neuroinflammatory process and PD progression in patients [75]. Further details of these biomarkers including sample source, summary of the study, and effects in the PD patients were taken into consideration and are given in Table 1. In addition, several biomarkers have limitations due to their specificity, as some of those mentioned overlap with clinical phenotypes of other such diseases including amyotrophic lateral sclerosis (ALS), neuropathy, frontotemporal dementia, and Alzheimer’s disease.

A few years back, multiple biomarkers for PD diagnosis were reported, and some of them are reliable and vital but still lack perfect accuracy. Usage of combined biomarkers will possibly improve disease diagnosis. Several studies have used the approach of combined biomarkers with clinical assessment and found it reliable to some extent [76,77]. So far, however, no biomarkers have provided enough accuracy and early-diagnosis ability, so this challenge may be compromised by using combined biomarkers that are region- and age-dependent.

**Table 1:** Neuroinflammatory biomarkers and their action in PD patients.

Inflammatory Biomarker		Sample Source	Summary	Effect in PD Patients	Reference
**Cytokines**	**IL-1β**	Peripheral blood/CSF	Sustained IL-1β expression in the striatum leads to DAergic neuronal death resulting in motor disabilities	IL-1β levels are elevated in the serum Probability of REM sleep behavior disorder (PRBD)	[69]
	**IL-2**	Blood serum	Blood IL-2 levels are decreased, which reduces the number and function of treg cells, affecting autoimmunity and causing lymphoproliferation	Higher serum IL-2 levels than control participants and occurrence of symptoms of depression or anxiety	[78]
	**IL-6**	CSF	Neuronal death in neurodegenerative diseases	Higher level of levels of plasma serum IL-6 that are correlated with an intense level of depression	[79]
	**IL-10**	CSF/serum	Develops neuroprotective effects against LPS-induced cell death	Higher peripheral level of IL-10	[80]
	**TNF-α/sTNFRs**	CSF	Activates microglia, which further induces the progressive loss of DAergic neurons	Positively associated with symptoms such as cognitive impairment, depression, and disability	[81]
	**NLRP3 (inflammasome protein)**	Biofluids	Progression biomarker in PD	Motor severity	[82]
**Chemokines**	**RANTES**	Blood serum	Proinflammatory chemokine involved in the regulation of immunoreactions and the addition of immune cells such as monocytes, granulocytes, and T cells to sites of inflammation	Elevated level in serum of PD patient, with serum RANTES levels positively correlated with H&Y stages and disease duration	[83]
**Protein**	**High-sensitivity C-reactive Protein (hsCRP)**	Plasma	Indicative marker of ongoing inflammation tissue damage	Serum hsCRP levels are elevated in patients with PD as compared to control participants	[84]
**Genetic mutation**	**Leucine-rich repeat kinase 2 (LRRK2)**	Familial and sporadic PD patients	Reduces the secretion of TNF-α, as its expression and kinase activity are upregulated in lipopolysaccharide (LPS)-activated microglia	Higher expression of IL-6 and TNFα	[85]
	**PTEN-induced putative Kinase 1 (PINK1)**	Familial PD patients	Detects mitochondrial dysfunction of phosphorylates in the *PARKIN* that degrade damaged mitochondria through a process called mitophagy	The upregulation of TNF-α, IL-1β, and IL-6 levels and mitophagy reduces inflammation by removing the damaged mitochondria	[72]
	**Parkin (PRKN)**	Autosomal-recessive early PD patients	Involved in mitophagy to degrade damaged mitochondria	Chronic inflammation	[86,87]
	**DJ-1**	Familial-recessive PD patients	Interrupts the role of protein in the regulation of membrane receptor tracing DJ-1, which connects to the p65 subunit of NFκB, and the knockdown of DJ-1, which directs p65 nuclear translocation	-	[74]
**Radiotracers**	**Translocater protein TSPO-1 ([11C]PK11195)**	PD patients	Elevated expression of mitochondrial translocator protein in activated microglia	Positively associated with a high level of motor dysfunction	[88,89]
	**TSPO-2 [18F]-FEPPA**	PD patients	Detects neuroinflammation that is specific to the striatum	-	[90,91]

## 5. Conclusions

The loss of DA neurons in the substantia nigra pars compacta (SNpc) is the hallmark of PD progression. Several genetic and environmental factors have been identified as major causes of PD; however, recent findings of glia dysfunction in PD have drawn attention. Little is known about the transition of resting microglia to activated microglia and resting astrocyte to reactive astrocyte. Neuroinflammation has been observed in PD patients; however, the major cause of such inflammation is still poorly understood. Furthermore, the major challenge is to diagnose PD in the early stage of its course for early intervention and better treatment. Early diagnosis of PD can be possible with the use neuroinflammation biomarkers. Tracing the alterations/modification of proinflammatory biomarkers including IL-1β, IL-6, IL-10, TNF-α, and RANTES in CSF or blood can be helpful in the early diagnosis, PD pathogenesis, and monitoring of disease progression. PD-associated genes are expressed in glial cells as well as in neurons and in noncell autonomous mechanisms. Any mutation or disruption in the expression of these PD-associated and PD-related genes leads to dysfunction in astrocytes and microglia. It is important to understand the pathogenic conditions in these glial cells that contribute to neuroinflammation and neuronal death. In the case of astrocytes, alpha-synuclein is clearly involved in the astrocyte-specific function including the increase and decrease of neurotrophic capacity and inflammatory responses [2]. Thus, more research is needed to understand the underlying causes of microglia and astrocyte-stimulated neurodegeneration, as well as to discover neuroinflammation markers. Although several neuroinflammation-based biomarkers have been identified, their accuracy is still lacking. A more timely and accurate diagnosis of PD will be possible with the identification of specific biomarkers based on the region, time, and age. Additionally, using a set of specific biomarkers together will improve sensitivity and accuracy of diagnosis and prognosis of the disease as well as assist in developing disease-modifying strategies.

## 6. Future Perspectives

Overall, targeting neuroinflammation biomarkers provides a significant breakthrough in understanding the etiology and pathogenesis of PD. Targeting inflammation in the peripheral region also seems promising, as the drugs do not necessarily cross the blood–brain barrier. Although contemporary research in PD is focused on personalized treatment, advancement in biomarker research is critical for robust identification of PD pathogenesis. Multimode or combined biomarkers usage would increase the specificity and accuracy of PD diagnosis, and exact and early diagnosis will help in designing potential therapeutic strategies. Understanding inflammation in PD will provide the pathological course and disease progression, and extensive research on neuroinflammation will provide specific biomarkers for early diagnosis of PD along with potential therapeutic targets for drugs development. There is focus on discovering multimodal markers of pathogenesis and PD progression with successful clinical trials, which will fill the gap in tailored treatment and disease-modifying options.

## Data Availability

No new data is generated from this study.
